# *BRCA*-associated hereditary male cancers: can gender affect the prevalence and spectrum of germline pathogenic variants?

**DOI:** 10.3389/fonc.2024.1414343

**Published:** 2024-06-21

**Authors:** Daniele Fanale, Lidia Rita Corsini, Chiara Brando, Ugo Randazzo, Marco Bono, Erika Pedone, Alessandro Perez, Roberta Sciacchitano, Daniela Cancelliere, Paola Piraino, Ambra Giurintano, Tancredi Didier Bazan Russo, Pietro Ferraro, Gaetana Rinaldi, Valeria Spinnato, Vincenzo Gennusa, Gianfranco Pernice, Salvatore Vieni, Gianni Pantuso, Antonio Russo, Viviana Bazan

**Affiliations:** ^1^ Section of Medical Oncology, Department of Precision Medicine in Medical, Surgical and Critical Care (Me.Pre.C.C.), University of Palermo, Palermo, Italy; ^2^ U.O. Oncologia, Fondazione Istituto G. Giglio, Cefalù, Palermo, Italy; ^3^ Division of General and Oncological Surgery, Department of Precision Medicine in Medical, Surgical and Critical Care (Me.Pre.C.C.), University of Palermo, Palermo, Italy; ^4^ Department of Biomedicine, Neuroscience and Advanced Diagnostics, University of Palermo, Palermo, Italy

**Keywords:** *BRCA1*, *BRCA2*, germline pathogenic variants, HBOC, male breast cancer, melanoma, pancreatic cancer, prostate cancer

## Abstract

**Introduction:**

Although hereditary male neoplasms are quite rare, individuals harbouring germline *BRCA1/2* pathogenic variants (PVs) may have a risk of developing tumours associated with Hereditary Breast and Ovarian Cancer (HBOC) syndrome, including male breast (MBC), prostate (PCa) and pancreatic (PC) cancers, and melanoma. Women and men showed a comparable genetic architecture of cancer susceptibility, but there are some gender-specific features. Since little is known about cancer genetic susceptibility in male population, our study was aimed at investigating the frequency of *BRCA1/2* PVs in men with HBOC syndrome-associated tumors, in order to understand whether differences in gender may reflect in the prevalence and spectrum of germline alterations.

**Patients and methods:**

We retrospectively collected and analysed clinical information of 352 HBOC-associated male cancer patients genetically tested for germline *BRCA1/2* PVs by Next-Generation Sequencing analysis, enrolled, from February 2018 to January 2024, at the “Regional Center for the prevention, diagnosis and treatment of rare and heredo-familial tumors of adults” of the University-Hospital Policlinico “P. Giaccone” of Palermo (Italy).

**Results:**

Our investigation revealed that 7.4% of patients was carrier of a germline *BRCA* PV, with an almost total prevalence of *BRCA2* alterations. In particular, 65.4% of *BRCA*-positive patients developed MBC, 19.2% had PC, 11.6% developed PCa, and only 3.8% had melanoma. Specifically, MBC individuals showed a *BRCA*-associated genetic predisposition in 17% of cases, whereas patients with PCa or PC exhibited a lower frequency of *BRCA2* PVs, taking into account the current national criteria for access to germline genetic testing.

**Discussion:**

Our study showed a high heterogeneity in prevalence of germline *BRCA2* PVs among men which could reflect a potential gender-specific genetic heterogeneity. Therefore, *BRCA*-associated male tumours could be due to *BRCA2* PVs different from those usually detected in women. In the event that it is demonstrated, in future, that male cancers are genetically distinct entities from those female this could improve personalized risk evaluation and guide therapeutic choices for patients of both sexes, in order to obtain a gender equality in cancer care.

## 1 Introduction

Germline pathogenic and likely pathogenic variants (herein called pathogenic variants, PVs) in the tumour suppressor genes *BRCA1* and *BRCA2* are predominantly associated with a significantly increased risk of breast and ovarian cancer in women, breast cancer (BC) and prostate cancer (PCa) in men, and pancreatic cancer (PC) and melanoma in both genders, with different risk rates (1). These *BRCA*-related tumours are associated with the hereditary breast and ovarian cancer (HBOC) syndrome, an inherited disorder which follows an autosomal dominant transmission mode (2).

Male breast cancer (MBC) is rare, accounting for about 1% of all BC diagnoses worldwide and less than 1% of cancers detected among men, with an increasing incidence (3, 4). In the general male population, the lifetime risk of developing BC is 0.1%, increasing up to 1.2% and 6.8% in the carriers of germline PVs in *BRCA*1 and *BRCA2*, respectively (5). Due to the extremely low incidence rate and the few conducted large-scale studies, current knowledge about MBC is low and treatment recommendations for male patients have largely followed those for postmenopausal women, even though MBC is a distinct tumour with different molecular and clinico-pathological features (6). Approximately 10% of all MBCs are hereditary tumours caused by germline alterations in well-defined BC susceptibility genes (7). Among these, *BRCA1*, and most commonly *BRCA2*, represent the most frequently involved high-penetrance genes, whose alterations are responsible for about 10–16% and 60–76% of hereditary MBC cases, respectively (8). The first multicenter study carried out in Italy showed a frequency of *BRCA1/2* PVs of about 13% (9).

*BRCA*-related MBCs have been shown to have specific molecular phenotypes and clinical characteristics (10). In particular, most *BRCA1*-related MBCs are grade 3, HER2-negative (HER2-) tumours with high proliferative activity, whereas *BRCA2*-associated MBCs are high-grade, HER2-positive (HER2+) tumours and show absence of progesterone receptor (PR) expression (7, 9).

PCa is the second most diagnosed cancer in men and the fifth leading cause of male cancer death, globally accounting for about 7% of newly diagnosed cases (11). Approximately 85% of new diagnoses involves individuals over 60 years of age (12).

PCa is a complex and heterogeneous neoplasm, classified as aggressive or non-aggressive, high-grade or low-grade, or early-onset (if occurring before age 55) or indolent (13). The PCa risk is 6% in the general population, while it rises up to about 9% in individuals harbouring *BRCA1* germline mutations and 15% in carriers of *BRCA2* PVs (5). PCa is diagnosed based on histologic subtype, location and Gleason score, which is currently the strongest prognostic factor (13).

Age, family history and genetic predisposition associated with germline *BRCA1/2* PVs have also been identified as important risk factors (14). In fact, the incidence of alterations in DNA damage repair defect (DDR) genes accounts for about 25% (15), with a higher prevalence in metastatic than localized disease, at about 12–16% and 5%, respectively (14). Inherited *BRCA2* PVs, most frequently detected in PCa, were found in about 3% of patients and were associated with early-onset, high-grade, greater aggressiveness and worse outcomes (15, 16).

PC is the 12th most frequent tumour and the sixth leading cause of death among men, with an incidence rate increasing by 0.5–1% per year and a death-to-incidence ratio of approximately 94% (11, 17). Most PCs are ductal adenocarcinomas (PDACs) of the exocrine pancreatic glands, accounting for about 85% of cases, frequently located in the head of the pancreas (18). Although they are predominantly of sporadic nature, a small percentage of PDAC is of familial origin and exhibits PVs which increase cancer genetic susceptibility. These alterations occur in *BRCA1/2* genes, predominantly in *BRCA2*, but also in mismatch repair genes, such as *MLH1*, *MSH2*, *MSH6* and *PMS2*, responsible for Lynch syndrome (19, 20).

In individuals with family history of cancer, 5–10% carries PVs in *BRCA2* gene and 1% in *BRCA1* with a risk of developing PC of 5 and 10%, respectively (16).

Another tumour which has been shown to be associated with germline *BRCA* alterations is melanoma. Although studies assessing the association between melanoma and *BRCA* are restricted and often inconclusive, *BRCA2* mutation carriers have been shown to have an increased risk of melanoma (21). The Breast Cancer Linkage Consortium study showed that carriers of *BRCA2* PVs were 2.5 times more likely to develop melanoma compared to the general population (22). Furthermore, also the studies by Moran et al. (23) and Johannsson et al. (24) confirmed an increased risk of melanoma in the presence of germline *BRCA2* PVs.

Several studies reported a significant heterogeneity in the prevalence of PVs across different populations. Because germline *BRCA* PVs confer an increased risk of different types of cancer and the prevalence of genetic alterations differs by ethnicity, race, gender, and different geographic location (25, 26), it could be interesting to investigate the prevalence of germline variants in Sicilian male patients affected by tumours included in the spectrum of the HBOC syndrome. For this purpose, in this work, we genetically tested Sicilian male patients with MBC, PCa, PC and melanoma for germline *BRCA1/2* PVs, in order to assess the type and prevalence of these high-risk susceptibility variants in individuals from the southernmost region of Italy.

## 2 Patients and methods

### 2.1 Study cohort

This retrospective cohort study was carried out from February 2018 to January 2024 at the “Sicilian Regional Centre for the Prevention, Diagnosis and Treatment of Rare and Hereditary Tumours” of the Medical Oncology Section of the “P. Giaccone” University Hospital in Palermo (Italy). The study involved 352 male cancer patients, including 100 consecutively recruited subjects with BC, 59 with PCa, 95 with PC and 98 with melanoma, taking into account the current national criteria for access to germline genetic testing. All patients, who had previously signed and accepted a written informed consent to study, were genetically tested for *BRCA1/2* PVs. The study (Protocol ‘G-Land 2017’) has been approved by the ethical committee (Comitato Etico Palermo 1; approval number: 0103–2017) of the university-affiliated hospital AOUP ‘P. Giaccone’ of Palermo. All relevant personal, family and clinical history information, including age at cancer diagnosis, histological subtype and stage of disease, was acquired and recorded anonymously for all patients, during a genetic counselling involving the presence of a multidisciplinary team made up of a geneticist, an oncologist, and a psychologist. The data on cancer diagnosis and histological subtype was recovered by pathology reports.

Following the genetic counselling for assessment of risk of HBOC syndrome, the patients were selected for germline *BRCA1/2* mutational screening based on probability rate of harbouring a PV, evaluated through the BRCAPRO genetic risk prediction model (27) and according to the criteria established by the Italian Association of Medical Oncology (AIOM) (https://www.aiom.it/raccomandazioni-per-limplementazione-del-test-brca-predittivo-e-preventivo-nei-tumori-della-mammella-dellovaio-del-pancreas-e-della-prostata/ and https://www.aiom.it/linee-guida-aiom-2023-melanoma/). These criteria, need to identify subjects at high risk of carrying a PV in the HBOC syndrome predisposition genes, rely on personal and family history of cancer and/or age at diagnosis. Specifically, on the basis of the different tumours included in the study, the selection of patients to be genetically tested for the search for germline *BRCA1/2* PVs was carried out using the following criteria: 1) MBC patients, regardless age of onset and family history of cancer; 2) metastatic PCa individuals, regardless age of onset and family history of cancer, and non-metastatic PCa patients with family history of no-Grade Group 1 PCa (according to the International Society of Urological Pathologists) diagnosed in at least one first-degree relative below age 60 years or in at least two family members below age 50 years (28); 3) metastatic/locally advanced PC individuals, regardless age at diagnosis and family history of cancer; 4) patients with personal history of synchronous/metachronous multiple melanoma (even in the absence of family history), melanoma patients with at least one affected first-degree relative in the same branch of the family, regardless age at diagnosis, and melanoma individuals with personal and/or family history of pancreatic adenocarcinoma.

The germline test result was considered informative when a pathogenic variant (PV) or likely pathogenic variant (LPV) was identified. Conversely, the test result was considered non-informative when no PV/LPV was identified, but its presence could not be excluded, or a variant of uncertain significance (VUS) was detected to which a risk value could not be attributed (29).

Carriers of a germline *BRCA1/2* PV have been subjected to intensive surveillance programs drawn up by an oncologist with expertise in cancer genetics. Targeted *BRCA1/2* testing has been proposed and extended to the first-degree family members of *BRCA*-mutated patients, after providing informed consent.

### 2.2 Sample collection and *BRCA1/2* genetic testing

Peripheral blood was collected from all patients included in the study. Genomic DNA was extracted from the peripheral leucocytes, using the DNeasy^®^ Blood Kit (QIAGEN, Hilden, Germany), and quantified by Qubit^®^3.0 fluorometer (Thermo Fisher Scientific, Waltham, MA). Its quality was assessed using 2100 Bioanalyzer (Agilent Technologies, Santa Clara, CA). The genetic analysis for identifying the *BRCA1/2* variants was carried out through next-generation sequencing (NGS) as previously described (26, 30). Furthermore, a Multiplex Ligation-dependent Probe Amplification (MLPA) analysis, performed as previously described (26, 30), was used to eventually detect the presence of large genomic rearrangements (LGRs) in *BRCA1* and *BRCA2* genes.

### 2.3 DNA Sanger sequencing

*BRCA1/2* PVs/LPVs were confirmed by Sanger sequencing using a BigDye Therminator 3.1 Cycle Sequencing Kit (Life Technologies, Carlsbad, CA, USA) and read through the 3130xl Genetic Analyzer (Applied Biosystems, Foster City, CA, USA), according to the manufacturers’ protocols.

### 2.4 Genetic variant classification

The detected genetic variants were classified based on the criteria developed by the Evidence-based Network for the Interpretation of Germline Mutant Alleles (ENIGMA) consortium (https://enigmaconsortium.org/) and according to the recommendations of the International Agency for Research on Cancer (IARC) (31). The classification involves a five-class division system: class I (benign variant), class II (probably benign variant), class III (VUS), class IV (probably PV), class V (PV). Several databases were used for the identification and classification of genetic variants, such as ClinVar, BRCA Exchange, LOVD and Varsome. The variants detected were denominated according to the recommendations for the description of sequence variants provided by the Human Genome Variation Society (HGVS). The HGVS nomenclature has been endorsed by HGVS, the Human Variome Project (HVP) and the Human Genome Organisation (HUGO) (32).

The localization of the germline variants on *BRCA1* and *BRCA2* genes detected in genetically tested patients was obtained and graphically represented using the informatic tool Mutation Mapper-cBioPortal for Cancer Genomics (33, 34).

## 3 Results

### 3.1 Clinico-pathological features of male cancer patients undergoing *BRCA1/2* genetic testing

Three hundred and fifty-two male cancer patients affected by MBC, PCa, PC and melanoma were recruited and studied over a period ranging from February 2018 to January 2024 at the “Regional Center for the prevention, diagnosis and treatment of rare and heredo-familial tumors of adults” of the Section of Medical Oncology of the University Hospital Policlinico “P. Giaccone” of Palermo (Italy). Among 352 recruited male individuals, 100 (28.4%) were affected by MBC, 59 (16.8%) by PCa, 95 (27%) by PC and 98 (27.8%) by melanoma.

One hundred MBC patients showed an average age at the diagnosis of 62 years. Considering the histological subtype, 9 (9%) out of 100 patients had *in situ* ductal carcinoma (DCIS), 70 (70%) invasive ductal carcinoma (IDC), 6 (6%) invasive lobular carcinoma (ILC) and, finally, 15 patients showed other types of BC. The most representative molecular phenotypes of MBC were luminal B/HER2- and luminal A (63% and 33%, respectively). Considering the family history, 20 patients (20%) had two or more family members affected by BC, 14 and 11 patients (14% and 11%, respectively) had a family history of ovarian cancer and PCa, respectively, and only 7 patients (7%) had one relative affected by PC. The clinical-pathological features of 100 MBC patients are summarized in **Table 1**.

**Table 1:** Clinical and pathological features of Male Breast Cancer (MBC) patients.

Characteristics of Patients (100)	No. of Patients (%)
Age at Diagnosis (years)	
< 50 years	15 (15)
≥50 years	85 (85)
**Average Age** (Range: 21–86)	62
Histological Subtype	
Invasive ductal	70 (70)
*In situ* ductal	9 (9)
Lobular	6 (6)
Others	15 (15)
Tumor Size (T)	
T1	56 (56)
T2	24 (24)
T3	11 (11)
T4	9 (9)
Axillary Nodal Involvement (N)	
N0	42 (42)
N1	30 (30)
N2	20 (20)
N3	8 (8)
Histologic Grade (G)	
G1	38 (38)
G2	45 (45)
G3	17 (17)
ER status	
Negative	17 (17)
Positive	83 (83)
PR status	
Negative	17 (17)
Positive	83 (83)
HER2 status	
Negative	58 (58)
Positive	42 (42)
Ki67 status	
<30%	44 (44)
>30%	56 (56)
Family History of Cancer	
Pancreas	7 (7)
Breast	20 (20)
Ovary	14 (14)
Prostate	11 (11)
Other	48 (48)
*BRCA1/2* Genetic Testing	
LPV/PV	17 (17)
VUS	3 (3)
Wild-Type	80 (80)

ER, Estrogen Receptor; LPV, Likely Pathogenic Variant; PR, Progesterone Receptor; PV, Pathogenic Variant; VUS, Variant of Uncertain Significance.

Considering the fifty-nine (16.8%) patients affected by PCa, the average age at diagnosis was 70 years, according to literature data (35). All patients presented a histological diagnosis of adenocarcinoma, poorly differentiated (G3) in 54% of cases, with a Gleason Score (GS) > 5 in 93% of individuals. Fourteen (24%) out of 59 patients showed a family history of PCa, whereas 13 (22%) subjects had at least one relative affected by BC (**Table 2**).

**Table 2:** Clinical and pathological features of Prostate Cancer (PCa) patients.

Characteristics of Patients (59)	No. of Patients (%)
Age at Diagnosis (years)	
< 50 years	1 (2)
≥50 years	58 (98)
**Average Age** (Range: 48–85)	70
Tumor Size (T)	
Tx	3 (5)
T1	10 (17)
T2	17 (29)
T3	19 (32)
T4	10 (17)
Nodal Involvement (N)	
Nx	11 (19)
N0	12 (20)
N1	19 (32)
N2	17 (29)
Metastasis (M)	
Mx	6 (10)
M0	0 (0)
M1	53 (90)
Histologic Grade (G)	
G1	11 (19)
G2	16 (27)
G3	32 (54)
Histology at Diagnosis	
Adenocarcinoma	59 (100)
Other	0 (0)
Gleason Score (GS)	
<5	4 (7)
>5	55 (93)
Family History of Cancer	
Pancreas	7 (12)
Breast	13 (22)
Ovary	6 (10)
Prostate	14 (24)
Other	19 (32)
*BRCA1/2* Genetic Testing	
LPV/PV	3 (5)
VUS	3 (5)
Wild-Type	53 (90)

LPV, Likely Pathogenic Variant; PV, Pathogenic Variant; VUS, Variant of Uncertain Significance.

The average age at diagnosis of 95 patients with PC was 63 years. In 42 (44%) individuals the tumor was localized in the pancreas head, whereas 30 (32%) and 23 (24%) patients had a tumor in the body and tail, respectively. At diagnosis time, 60 (63%) patients had a metastatic tumor and 35 (37%) a locally advanced cancer. Ten (11%) out of 95 patients had a family history of PC, 11 (12%) subjects had at least a relative with BC, and 12 (13%) showed a positive family history for PCa (**Table 3**).

**Table 3:** Clinical and pathological features of Pancreatic Cancer (PC) patients.

Characteristics of patients (95)	No. of patients (%)
Age at diagnosis (years)	
< 50 years	10 (11)
≥50 years	85 (89)
**Average Age** (Range: 31–85)	63
Tumor Location	
Head	42 (44)
Body	30 (32)
Tail	23 (24)
Tumor Size (T)	
Tx	2 (2)
T1	3 (3)
T2	23 (24)
T3	30 (32)
T4	37 (39)
Nodal Involvement (N)	
Nx	17 (18)
N0	12 (13)
N1	32 (33)
N2	34 (36)
N3	0 (0)
M Status	
Mx	13 (14)
M0	22 (23)
M1	60 (63)
Histological Grade (G)	
G1	4 (4)
G2	38 (40)
G3	53 (56)
Tumor Status at Diagnosis	
Locally advanced	35 (37)
Metastatic	60 (63)
Family History of Cancer	
Pancreas	10 (11)
Breast	11 (12)
Ovary	6 (6)
Prostate	12 (13)
Other	56 (58)
*BRCA1/2* Genetic Testing	
LPV/PV	5 (5)
VUS	3 (3)
Wild-type	87 (92)

LPV, Likely Pathogenic Variant; PV, Pathogenic Variant; VUS, Variant of Uncertain Significance.

Finally, the average age at diagnosis of 98 investigated patients with melanoma was 48 years. In 64 (65%) out of 98 patients the primary tumors were mainly located in the trunk, whereas 25 (26%) individuals presented melanomas in limbs and 9 (9%) patients in head and neck region. The most frequent histological tumor subtypes observed in patients enrolled in this study were superficial spreading melanoma (67%) followed by *in situ* and nodular melanoma (28% and 5%, respectively). Ten patients (10%) had a family history of PCa, whereas 9 (9%) and 8 (8%) patients had a positive family history for PC and BC, respectively (**Table 4**).

**Table 4:** Clinical and pathological features of melanoma patients.

Characteristics of Patients (98)	No. of Patients (%)
Age at Diagnosis	
<50 years	53 (54)
≥50 years	45 (46)
**Average Age (**Range: 18–92)	48
Site of Primary MM	
Head and Neck	9 (9)
Trunk	64 (65)
Limbs	25 (26)
Histological Subtype	
SSM	66 (67)
NM	5 (5)
*In Situ*	27 (28)
No. of Primary MM	
Single	52 (53)
Multiple Synchronous Metachronous	46 (47) 9 37
Breslow Thickness	
*In situ*	29 (30)
< 1mm	49 (50)
1–2 mm	13 (13)
>2 mm	7 (7)
AJCC Stage^*^	
0	16 (17)
Ia	39 (40)
Ib	10 (10)
IIa	13 (13)
IIb	5 (5)
III	10 (10)
IV	5 (5)
Family History of Cancer	
Pancreas	9 (9)
Breast	8 (8)
Ovary	0 (0)
Prostate	10 (10)
Other	71 (73)
*BRCA1/2* Genetic Testing	
LPV/PV	1 (1)
VUS	0 (0)
Wild-Type	97 (99)

^*^Disease stage was defined according to the recent American Joint Committee on Cancer (AJCC) guidelines.

MM, Malignant Melanoma; SSM, Superficial Spreading Melanoma; NM, Nodular Melanoma; LPV, Likely Pathogenic Variant; PV, Pathogenic Variant; VUS, Variant of Uncertain Significance.

### 3.2 Germline *BRCA1/2* pathogenic variants in male patients with HBOC syndrome-associated tumours

**Figure 1:**
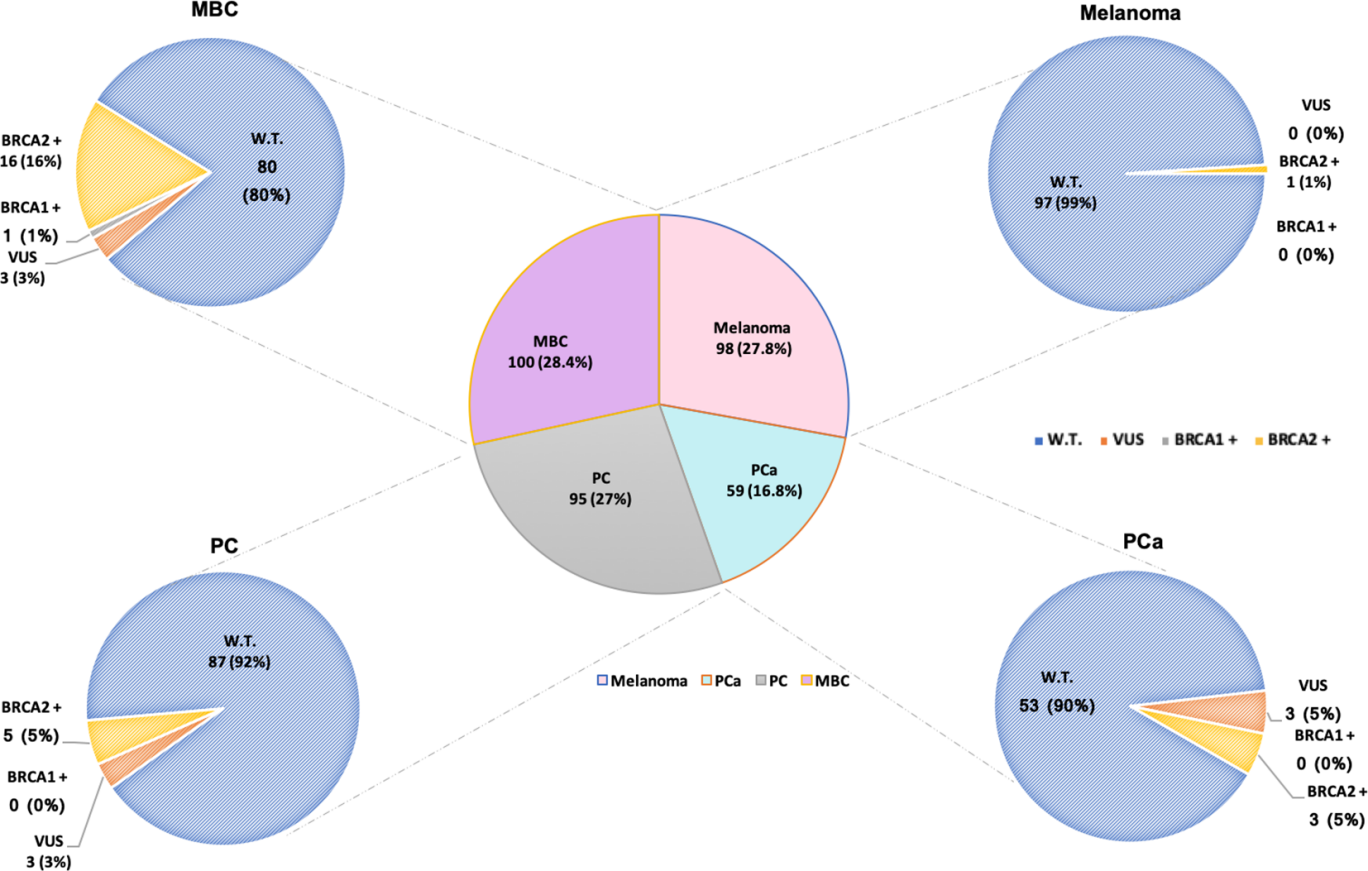
Number of male patients affected by HBOC syndrome-associated tumors genetically tested for germline *BRCA1/2* variants. Total number of analyzed patients, on the basis of the different tumors, is divided into carriers of *BRCA1/2 wild-type* (blue), variants of uncertain significance (VUS) in *BRCA1/2* (orange), and pathogenic/likely pathogenic variants (PVs/LPVs) in *BRCA1* (grey) and *BRCA2* (yellow) genes, respectively. Patients harboring benign/likely benign variants (BVs/LBVs) are considered carriers of *BRCA1/2 wild-type*.

All 352 male probands with HBOC syndrome-associated tumours, after appropriate genetic counselling, aimed at ascertaining the criteria concerning personal and family history of cancer recommended by the AIOM national guidelines, were genetically tested for germline variants in *BRCA1* and *BRCA2* genes. The mutational screening of the investigated study population showed that 26 (7.4%) out of 352 patients with MBC, PCa, PC or melanoma harboured germline *BRCA* PVs (*BRCA*-positive), whereas 9 (2.6%) probands were carriers of germline *BRCA1/2* VUS (class III), and 317 (90%) subjects carried a germline *BRCA1/2* benign/likely benign variants (*BRCA*-*w*.*t*.) (**Figure 1**). No LGR in *BRCA1/2* genes was detected in examined study cohort.

In particular, our genetic analysis showed that 17 (65.4%) out of 26 male individuals positively tested for *BRCA* PVs had MBC, whereas 5 (19.2%) were affected by PC, 3 (11.6%) showed PCa and only 1 (3.8%) had melanoma (**Table 5**). Based on the classification criteria developed by the ENIGMA consortium and according to the IARC recommendations (31), the mutational analysis revealed the presence of 20 different *BRCA* PVs, 19 of which in *BRCA2* and only one in *BRCA1*, detected in 26 tested patients. The *BRCA2* variant named c.4284dup (p.Gln1429fs) has been observed in both melanoma and MBC, whereas another *BRCA2* variant named c.7681C>T (p.Gln2561Ter) was present in one individual with MBC and one with PC (**Table 5**). Both *BRCA2* variants have been previously detected with low frequency rates (0.82% and 1.03%, respectively) in women of the same population affected by breast and/or ovarian cancer (26).

**Table 5:** Germline *BRCA* PVs/LPVs harbored by patients with MBC, PCa, PC or melanoma.

Gene	Variant Type	HGVS Nomenclature	Protein change	Variant Interpretation	No. patients
Breast Cancer (BC)					
***BRCA2***	fs	c.6078_6079del	p.Glu2028fs	V	2
***BRCA2***	fs	c.9026_9030del	p.Tyr3009fs	V	2
***BRCA2***	NS	c.1842dup	p.Asn615Ter	V	2
***BRCA2***	fs	c.1238del	p.Leu413Hisfs	V	2
***BRCA2***	fs	c.1472del	p.Thr481Ilefs18	V	1
***BRCA2***	fs	c.4284dup	p.Gln1429fs	V	1
***BRCA2***	fs	c.6082_6086del	p.Glu2028LysfsTer19	V	1
***BRCA2***	missense	c.631G>A	p.Val211Ile	V	1
***BRCA2***	missense	c.7007G>A	p.Arg2336His	V	1
***BRCA2***	NS	c.7681C>T	p.Gln2561Ter	V	1
***BRCA2***	NS	c.7913_7917del	p.Ala2637_Phe2638insTer	V	1
***BRCA2***	NS	c.8594T>A	p.Leu2865Ter	V	1
***BRCA1***	NS	c.4327C>T	p.Arg1443Ter	V	1
Prostate Cancer (PCa)					
***BRCA2***	NS	c.3545_3546del	p.Gln1181_Phe1182insTer	V	1
***BRCA2***	NS	c.8969G>A	p.Trp2990Ter	V	1
***BRCA2***	fs	c.1813del	p.Ile605fs	V	1
Pancreatic cancer (PC)					
***BRCA2***	IVS	c.8487 + 1G>A	/	V	1
***BRCA2***	IVS	c.1909 + 1G>A	/	V	1
***BRCA2***	NS	c.7681C>T	p.Gln2561Ter	V	1
***BRCA2***	missense	c.9302T>C	p.Leu3101Pro	IV	1
***BRCA2***	IVS	c.476–2A>G	/	V	1
Melanoma					
***BRCA2***	fs	c.4284dup	p.Gln1429fs	V	1

HGVS, Human Genome Variation Society; IVS, Intronic Variant Sequence, NS, Nonsense; fs, frameshift.

However, no variant in particular has shown high prevalence within the examined male population.

As regards the typology of germline *BRCA* PVs observed in our study population, approximately two-third of the variants were frameshift (7) and nonsense (7), whereas about one-third were intronic variant sequences (IVS, 3) and missense (3). All IVSs were detected only in men affected by PCa, while almost all found frameshift variants were present in MBC patients (**Table 5**).

Furthermore, we investigated the presence of VUS and other variants with conflicting interpretations of pathogenicity (CIP) in *BRCA1/2* genes of 352 individuals, identifying 9 different VUS/CIP (4 in *BRCA1* and 5 in *BRCA2*) in 9 patients, 3 of which had MBC, 3 showed PCa, and 3 were affected by PC. No germline *BRCA1/2* VUS/CIP was detected in melanoma patients (**Table 6**).

**Table 6:** Germline *BRCA* variants of uncertain significance harbored by MBC, PCa or PC patients.

Gene	Variant Type	HGVS Nomenclature	Protein change	Variant Interpretation	No. patients
Breast Cancer patients					
***BRCA1***	missense	c.1561G>A	p.Ala521Thr	CIP	1
***BRCA1***	missense	c.3394A>G	p.Asn1132Asp	CIP	1
***BRCA1***	missense	c.4739C>T	p.Ser1580Phe	VUS	1
Prostate Cancer patients (PCa)					
***BRCA1***	missense	c.889A>C	p.Met297Leu	CIP	1
***BRCA2***	missense	c.9116C>T	p.Pro3039Leu	CIP	1
***BRCA2***	missense	c.31T>G	p.Phe11Val	VUS	1
Pancreatic cancer patients (PC)					
***BRCA2***	missense	c.9898C>T	p.Pro3300Ser	VUS	1
***BRCA2***	IVS	c.6842–23delAT	/	CIP	1
***BRCA2***	missense	c.5669T>C	p.Met1890Thr	CIP	1

CIP, Conflicting Interpretations of Pathogenicity​; HGVS, Human Genome Variation Society; IVS, Intronic Variant Sequence; VUS, Variant of Uncertain Significance.

Considering a distinction for neoplasm, among the 100 MBC patients, 16 were carriers of germline PVs in *BRCA2* and only one in *BRCA1*, whereas 80 were *BRCA1/2*-*wild-type* (**Figure 1**). Specifically, 12 different PVs have been identified in *BRCA2* gene and only one in *BRCA1* gene. The *BRCA2* variants named c.6078_6079del (p.Glu2028fs), c.9026_9030del (p.Tyr3009fs), c.1842dup (p.Asn615Ter) and c.1238del (p.Leu413Hisfs) have been detected each in two different MBC patients (**Table 5**).

Among 59 PCa patients, 53 (90%) showed no genetic susceptibility related to *BRCA* alterations (*BRCA1/2*-*wild-type*), whereas three men (5%) have been shown to be carriers of a germline PV in *BRCA2* gene (**Figure 1**). All three *BRCA2*-mutated patients showed advanced disease and a significant family history of cancers associated with the HBOC syndrome. In particular, one of them had two first-degree relatives affected by PCa, whereas the other two showed a family history of female BC in three first-degree relatives (mother and sisters). No germline alteration was detected in *BRCA1* gene. Likewise, in almost all men affected by PC (87/95, 92%) no *BRCA*-associated genetic predisposition was found, except in 5 (5%) patients with family history of cancer who harboured germline PVs only in *BRCA2* gene. As regards the individuals with melanoma, only one out of 98 analyzed patients was carrier of a germline *BRCA2* PV (**Figure 1**), showing a family history of female BC in two first-degree relatives (mother and sister).

**Figure 2:**
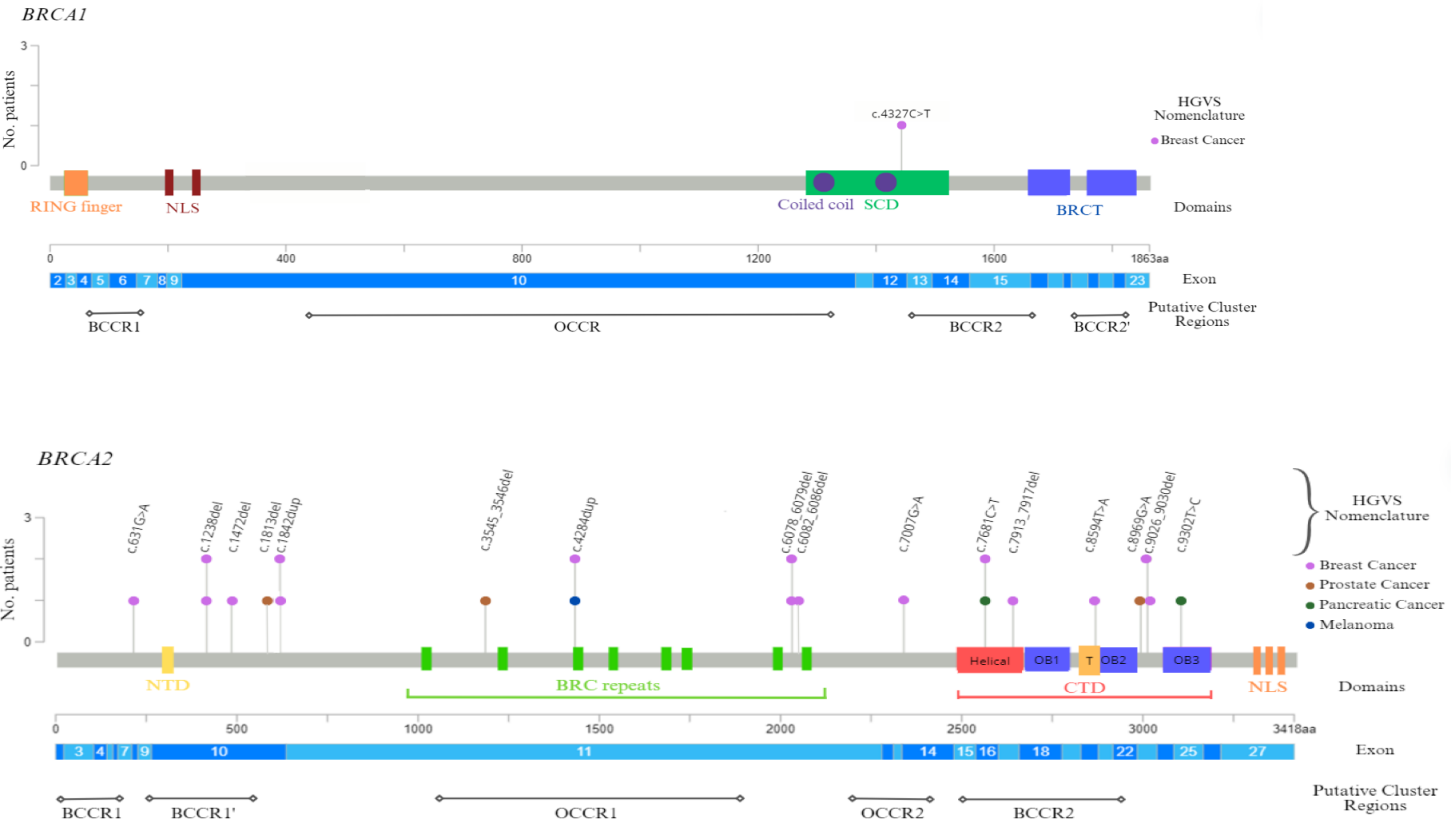
BRCA1 and BRCA2 functional domains and gene location of germline *BRCA1/2* pathogenic variants detected in male cancer patients. The lollipop plots show the distribution and frequency of *BRCA1/2* PVs identified in study population. The plots were obtained by the informatic tool Mutation Mapper-cBioPortal for Cancer Genomics (GenBank Reference *BRCA1*: NM_007294 and GenBank Reference *BRCA2*: NM_000059). The lollipop height indicates the frequency of *BRCA1/2* PVs. BCCR, Breast Cancer Cluster Region; BRCT, BRCA1 C-terminus domain; CTD, C-terminal DNA-binding domain; HGVS, Human Genome Variation Society; NLS, nuclear localization sequence; NTD, N-terminal DNA-binding domain; OB, oligonucleotide binding; OCCR, Ovarian Cancer Cluster Region; SCD, serine cluster domain; T, Tower region.

Finally, our study also analyzed gene location of the germline *BRCA* PVs, in order to investigate eventual associations between specific variants and tumor phenotype, since several studies suggested a strong correlation between specific *BRCA1/2* variants and changes in breast and ovarian cancer relative risk, by identifying specific putative Breast Cancer Cluster Regions (BCCRs) and Ovarian Cancer Cluster Regions (OCCRs), located on the coding DNA sequences of *BRCA1* and *BRCA2* genes (36–39). All *BRCA2* variants detected in male cancer patients have been observed to be distributed along the entire *BRCA2* gene sequence (**Figure 2**). However, in the case of MBC patients, half of these alterations (6/12) was mainly localized inside two putative cluster regions, BCCR1’ and BCCR2, present in the BRCA2 protein structure, near the N-terminus and C-terminus, respectively. Specifically, three *BRCA2* variants were located in the N-terminal BCCR1’ region (nucleotides: 1238–1842; codons: 413–615), included inside the exon 10, whereas four variants (nucleotides: 7681–9030; codons: 2561–3009) were detected in the DNA sequence corresponding to the C-terminal DNA-binding domain (CTD), which includes the putative BCCR2 region. Additionally, other three *BRCA2* PVs were located in the DNA sequence corresponding to the “BRC repeats” domain (nucleotides: 4284–6086; codons: 1429–2028), included within the exon 11 (**Figure 2**). Therefore, a correlation between the variant localization in the BCCRs of *BRCA2* and type of tumor was observed in 50% (8/16) of *BRCA2*-positive MBC patients.

## 4 Discussion

To date, extensive research on the risk of hereditary cancer and prevalence of *BRCA1/2* PVs in women was performed, whereas little is known about this in men harboring germline *BRCA1/2* alterations. Although women and men share a similar genetic architecture of cancer susceptibility, there are some sex-specific pathological features observed in BC (40, 41) (42) and potential additional differences that are currently being investigated by the CONFLUENCE project. Therefore, considering male cancers as genetically distinct entities from those female could improve the evaluation of personalized risk and drive therapeutic choices of patients of both sexes, with the purpose to obtain a gender equality in cancer care (43). Beyond the preventive significance for probands and family members, the *BRCA1/2* genetic testing has recently assumed also a predictive value of response to specific biological therapies in patients affected by PCa or PC (44).

Previous results (26) regarding women from the same geographical area affected by breast and/or ovarian cancers showed a relatively lower frequency rate of *BRCA1/2* PVs (14.8%, 200 out of 1346 probands) compared to males with BC investigated in our study (17%, 17 out of 100 probands). However, this data regards population cohorts different in numerical terms and by neoplasm, as ovarian cancer women have been also included in the studied population.

A significant heterogeneity in the prevalence of *BRCA* PVs across different populations was reported by several studies. Based on these data, the inherited cancer risk estimate could also be influenced by race and ethnic origin, beside gender (26, 43, 45). As already reported in other studies, the elaboration of new population-based genetic approaches may help to identify the 50% more *BRCA* PV carriers than those detected by conventional clinical and familial criteria. Therefore, population-based genetic information, in the future, could be useful to improve the detection strategies of *BRCA1/2* PV carriers and maximize prevention paths, with significant implications for clinical management and surveillance of male cancer patients and their family members (46).

Since little is known about genetic susceptibility to cancer in Sicilian male population and data on germline variant frequency is limited or conflicting, our study was aimed at investigating the prevalence and type of inherited *BRCA1/2* PVs in 352 men from a specific geographical area of Southern Italy. Our results showed that about 7% of men who met the current criteria for genetic testing were *BRCA* PV carriers. The majority of these were *BRCA2* mutations (25 out of 26 patients), which is consistent with previous reported data (1, 47). Whereas different population-based studies often reported variable and non-uniform data about the frequency of *BRCA2* PVs in MBC cases, showing percentages ranging between 6.8% and 16% (40, 42, 47), compared to the rest of the Italian population, our results support a higher *BRCA* mutation rate in Sicilian men. In contrast, both PCa and PC had similar mutation rates to those reported in the literature (15, 16). As recently highlighted by Li and colleagues (44), our analysis showed a very weak association between melanoma risk and germline *BRCA2* alterations (only one individual out of 98 patients). In contrast, other studies either found no association between *BRCA2* PV and melanoma risk or showed a moderate increase in risk compared with the general population (48–51). Globally, our results showed that there is a high heterogeneity of germline *BRCA2* PVs among individuals of our study cohort, as only a very few specific variants were shared between patients.

No variant in particular has been observed with high prevalence within the examined male population. The most common Sicilian founder mutation named *BRCA1–*5083del19 (HGVS nomenclature: c.4964_4982del; p.Ser1655fs) (26, 52, 53), usually detected with high frequency in women with HBOC syndrome, was not found in the examined male population affected by HBOC syndrome-associated tumours. In addition, the most recurrent *BRCA2* PV in Sicilian female population with HBOC (26), named 1466delT (HGVS nomenclature: c.1238del; p.Leu413fs), has been observed with very low frequency in men affected by MBC, PCa, PC or melanoma. The heterogeneous distribution of germline *BRCA* PVs and the lack of a specific territorial prevalence of these variants could reflect the genetic heterogeneity of the populations belonging to regions of Southern Italy and their historical background due to the different colonisations as well as to the crucial geographical localization of Sicily in the centre of Mediterranean Sea, crossroads of several ethnicities and cultures throughout history (54, 55). We previously observed a higher prevalence of some germline *BRCA* PVs in the Sicilian female population affected by hereditary breast or ovarian cancers which suggested the possibility of a population-specific genetic signature (26). This study, instead, showed the absence of specific genetic features in male population affected by *BRCA*-related tumors. As soon as the data from the CONFLUENCE project become available, probably it will be possible to better deep this hypothesis. A higher frequency of some germline *BRCA1/2* PVs may be associated, not only with a particular ethnicity, but also with a specific gender. This suggests that those variants may be female-specific.

Knowing the genetic background underlying the phenotype of each tumor may have not only prognostic, but also preventive and therapeutic implications.

## Data availability statement

All relevant data is contained within the article. The datasets presented in this article are not readily available because of ethical and privacy reasons. Requests to access the datasets should be directed to antonio.russo@usa.net and viviana.bazan@unipa.it.

## Ethics statement

The studies involving humans were approved by Comitato Etico Palermo 1 (approval number: 0103-2017) of the University-affiliated Hospital A.O.U.P. ‘P. Giaccone’ of Palermo. The studies were conducted in accordance with the local legislation and institutional requirements. The participants provided their written informed consent to participate in this study.

## Author contributions

DF: Conceptualization, Data curation, Formal analysis, Funding acquisition, Investigation, Methodology, Project administration, Supervision, Validation, Writing – original draft, Writing – review & editing. LC: Conceptualization, Data curation, Formal analysis, Funding acquisition, Investigation, Supervision, Validation, Writing – original draft, Writing – review & editing. CB: Conceptualization, Data curation, Formal analysis, Investigation, Methodology, Supervision, Writing – original draft, Writing – review & editing. UR: Conceptualization, Data curation, Formal analysis, Investigation, Supervision, Validation, Writing – original draft, Writing – review & editing. MB: Data curation, Methodology, Writing – review & editing. EP: Data curation, Methodology, Writing – review & editing. AP: Data curation, Methodology, Writing – review & editing. RS: Data curation, Formal analysis, Writing – review & editing. DC: Data curation, Methodology, Writing – review & editing. PP: Writing – review & editing, Data curation, Formal analysis. AG: Data curation, Methodology, Writing – review & editing. TB: Data curation, Methodology, Writing – review & editing. PF: Data curation, Methodology, Writing – review & editing. GR: Methodology, Writing – review & editing. VS: Data curation, Methodology, Writing – review & editing. VG: Data curation, Formal analysis, Methodology, Writing – review & editing. GFP: Formal analysis, Methodology, Writing – review & editing. SV: Data curation, Methodology, Validation, Writing – review & editing. GP: Data curation, Methodology, Validation, Writing – review & editing. AR: Data curation, Investigation, Methodology, Project administration, Supervision, Validation, Writing – review & editing. VB: Formal analysis, Investigation, Methodology, Project administration, Supervision, Validation, Writing – review & editing.
